# Cutaneous wound healing: recruiting developmental pathways for regeneration

**DOI:** 10.1007/s00018-012-1152-9

**Published:** 2012-10-04

**Authors:** Kirsten A. Bielefeld, Saeid Amini-Nik, Benjamin A. Alman

**Affiliations:** 1 Program in Developmental and Stem Cell Biology, Department of Developmental and Stem Cell Biology, Hospital for Sick Children Research Institute, Toronto Medical Discovery Tower, East Tower, 101 College St., Toronto, ON M5G 1L7 Canada; 2 Department of Laboratory Medicine and Pathobiology, University of Toronto, Toronto, ON M5S 1A8 Canada; 3 Department of Surgery, University of Toronto, Toronto, ON M5S 1A8 Canada

**Keywords:** Wound healing, Regeneration, Skin, Wnt, β-Catenin, Transforming growth factor β (TGF-β), Notch, Hedgehog

## Abstract

Following a skin injury, the damaged tissue is repaired through the coordinated biological actions that constitute the cutaneous healing response. In mammals, repaired skin is not identical to intact uninjured skin, however, and this disparity may be caused by differences in the mechanisms that regulate postnatal cutaneous wound repair compared to embryonic skin development. Improving our understanding of the molecular pathways that are involved in these processes is essential to generate new therapies for wound healing complications. Here we focus on the roles of several key developmental signaling pathways (Wnt/β-catenin, TGF-β, Hedgehog, Notch) in mammalian cutaneous wound repair, and compare this to their function in skin development. We discuss the varying responses to cutaneous injury across the taxa, ranging from complete regeneration to scar tissue formation. Finally, we outline how research into the role of developmental pathways during skin repair has contributed to current wound therapies, and holds potential for the development of more effective treatments.

## Cutaneous wund healing and skin development

Cutaneous wound repair recapitulates embryonic skin development in numerous aspects, in an attempt to restore the integrity of the injured tissue. Both processes involve the differentiation, migration, proliferation, and apoptosis of various cell types to create the multilayered tissue that constitutes the skin. Many of the same key signaling pathways that are activated during embryonic skin development are also activated during postnatal cutaneous wound repair; these include the Wnt/β-catenin, Notch, Hedgehog, and various growth factor/cytokine pathways. Furthermore, several ‘embryonic’ extracellular matrix (ECM) components, such as Extra-Domain-A (EDA) fibronectin, are synthesized during postnatal wound repair [1, 2]. Despite these similarities, there are a number of important differences between the molecular mechanisms that regulate postnatal cutaneous wound repair and embryonic skin development, and these may partly be responsible for the inability of repaired skin to achieve its original uninjured state.

 Repaired skin, which usually heals as a scar, is weaker than intact skin, and contains a disorganized ECM compared to nonwounded skin, and healing early gestational fetal wounds [3–6]. Cutaneous wounds do not normally show regeneration of hair follicles, although an exception has been documented in the case of large cutaneous wounds [7]. As a result, postnatal mammalian skin repair is not identical to the process of regeneration, in which the regenerated tissue is almost indistinguishable from the uninjured tissue [5, 8]. Part of the reason for this difference is the inflammatory response, which is unique to postnatal wound healing [4, 9]. While the inflammatory response is crucial to protect the body from invading foreign organisms at the injury site, many of the inflammatory cytokines and growth factors released during this process promote fibrosis and scar formation [10, 11]. Indeed, embryonic wounds tend to heal without scarring, and it is believed that this is due to the relative lack of an inflammatory response caused by the absence of a fully developed immune system [4, 5, 9]. Though early fetal healing does incorporate growth factors and cytokines, the expression profiles and concentrations of these molecules are different from those in scar-forming late gestational and adult healing [4, 6, 9]. For example, scarless fetal wound healing is characterized by lower levels of transforming growth factor-β1 (TGF-β1), and higher concentrations of TGF-β3, compared to scar-forming wounds [6, 9]. Similarly, the composition and/or levels of certain ECM components, such as hyaluronic acid, fibronectin, and elastin, differ in fetal versus postnatal skin [9, 12, 13], and may influence the healing outcome.

 Additional insight into the mechanisms that cause embryonic skin development and repair to differ from postnatal mammalian skin healing are being elucidated by studies of organisms such as amphibians, which regenerate their injured tissue in a process analogous to development [5, 8]. Enhancing our understanding of the molecular pathways that are responsible for these differences is vital for generating novel medical therapies to improve wound healing and reduce scarring. Here, we discuss the role of developmental signaling pathways in cutaneous wound repair, with an emphasis on keratinocyte and fibroblast behavior, and compare and contrast this with their roles in skin development. We also outline the varying responses to injury across the taxa, ranging from complete regeneration to scar tissue formation. Finally, we discuss current clinical applications that may improve wound healing via the modulation of developmental pathways, and map out future areas of research which remain to be addressed.

## Stages of cutaneous wound healing

**Fig. 1:**
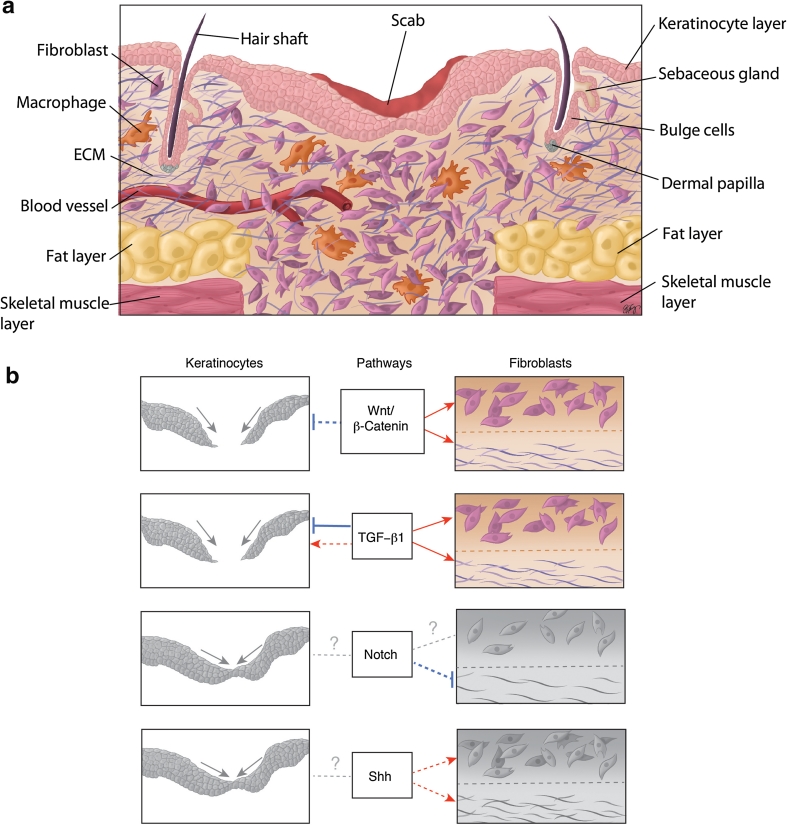
Proliferative phase of murine cutaneous wound healing. **a** Illustrative histological section of a murine cutaneous wound during the proliferative phase of repair. Healing dermis is enriched with higher numbers of fibroblasts and macrophages compared to intact skin. **b** The effect of the developmental signaling pathways on keratinocyte behavior in epidermal closure, and fibroblast behavior and matrix deposition in dermal reconstitution, respectively, is depicted. *Red arrows* indicate a positive or stimulatory effect of a pathway on a cell type/outcome. *Blue* “inhibitory” symbols indicate an inhibitory effect. *Solid lines* indicate that the effect of a pathway on each cell type and/or outcome is supported by substantial in vivo evidence in the literature. *Dotted lines* indicate effects which either lack sufficient in vivo evidence or are based mainly on in vitro work. *Dotted gray lines with a question mark* indicate unknown or unclear outcomes. *Colored diagrams* represent outcomes of pathways in each cell type (or matrix deposition) that are supported by substantial in vivo evidence in the literature. In contrast, *gray diagrams* represent outcomes that are based mainly on in vitro evidence or require further in vivo investigation; *Gray diagrams* linked by a *simple dotted line* indicate that there is either no effect, or that the effect is not known. Refer to the text for a detailed explanation of the effect of each signaling pathway on keratinocyte and fibroblast behavior during wound repair

The skin is composed of two main layers: the superficial layer, the epidermis, which functions as a barrier to the external environment, and the deeper layer, the dermis, which is composed of connective tissue, and provides the skin with its mechanical properties. The epidermis consists of a stratified keratinized epithelium that is interspersed with hair follicles and glands [14–16]. Underlying the epidermis is the dermis, subdivided into the upper ‘papillary’ dermis, and the lower ‘reticular’ dermis, which differ in the density of their collagen fibers [16]. During cutaneous wound healing, the barrier and mechanical properties of skin are restored by the actions of numerous cell types which undergo proliferation, differentiation, migration and apoptosis to rebuild the skin. Normal cutaneous wound repair is characterized by three overlapping phases of healing termed the inflammatory, proliferative, and remodeling phases [3, 5, 14, 15] (Table 1; Fig. 1a).

**Table 1:** Summary of the stages of wound repair

Stage of healing	Main processes	References
Hemostasis and inflammation	Hemostasis	[3, 15, 17–19]
	Vasoconstriction	
	Formation of fibrin clot	
	Inflammation	
	Release of cytokines and growth factors by platelets and immune cells, and from the disrupted matrix	
	Invasion of inflammatory cells (neutrophils, monocytes-macrophages)	
Proliferation	Dermis	[1–3, 5, 15, 20, 23, 25, 27, 28, 30]
	Release of growth factors by macrophages and fibroblasts	
	Fibroblast migration and proliferation	
	Synthesis of matrix proteins (fibronectin and collagen)	
	Angiogenesis	
	Epidermis	
	Keratinocyte migration, proliferation and differentiation	
	Contributions from hair follicle stem cells	
	Possible contribution from interfollicular epidermal stem cells	
Remodeling	Reorganization and remodeling of the ECM	[3, 5, 18, 20, 32, 33]
	Myofibroblast formation	
	Contraction of the wound	
	Cell apoptosis

### Hemostasis and inflammation

Hemostasis forms the immediate response to injury and functions to prevent the loss of blood at the wound site. Vascular injury initiates a cascade of events that terminates in coagulation, and encompasses vascular constriction, platelet aggregation and degranulation, and finally the formation of a fibrin clot [3, 17, 18]. The fibrin clot also acts as a provisional matrix for the initial migration of inflammatory cells to the wound site [3, 17, 18]. Inflammatory cells, such as neutrophils and monocytes, are attracted to the site of injury by cytokines, including TGF-β and platelet-derived growth factor (PDGF), which are released by platelets and from sites of sequestration in the disrupted ECM [15, 17]. Neutrophils remove bacteria and/or foreign objects from the wound and are followed by monocytes, which subsequently differentiate into macrophages [19]. While macrophages phagocytose foreign organisms, particles, and dead neutrophils, they also release TGF-β and other cytokines, and thereby stimulate the movement of fibroblasts and epithelial cells into the wound [15, 19].

### Proliferation

The proliferative phase of wound healing (Fig. 1a) is characterized by re-epithelialization of the epidermis, and by repair of the underlying dermal or mesenchymal layer. This is accompanied by neovascularization. The dermis is restored by invading and proliferating fibroblasts that synthesize and secrete ECM proteins and also release activating growth factors such as TGF-β1 [3, 11, 20]. During the proliferative phase of wound repair, fibroblasts produce immature or ‘embryonic’ ECM variants such as EDA fibronectin and type III collagen, as well as the collagen type I that is normally found in adult skin [1–3, 20]. The invasion of fibroblasts into the wound is facilitated by their secretion of ECM-cleaving matrix metalloproteinases (MMPs) [15]. Epidermal keratinocytes express various integrin receptors during wound repair and are thought to use the provisional matrix as a substrate for re-epithelialization [21, 22]. Closure of the epithelial gap and restoration of the epithelium is important as a barrier function, and this is achieved by a combination of keratinocyte migration, proliferation and differentiation [5, 23]. Different populations of hair follicle stem cells [24–26], both inside [27] and outside of [28] the hair follicle bulge region, contribute to re-epithelialization during wound repair. Interfollicular epidermal stem cells [24, 29] may also participate in this process. For example, Langton et al. [30] found that the tail skin of wounded mutant mice that lack hair follicles still exhibited (albeit delayed) re-epithelialization. However, further research is needed to unravel the details of a potential contribution by interfollicular stem cells during wound healing.

### Remodeling

The remodeling of the wound site, following deposition of sufficient ECM and the closure of the epithelial gap, changes the properties of the tissue. Wound fibroblasts at this stage of the repair process tend to adopt a contractile myofibroblast phenotype [31, 32]. Reorganization of the wound tissue involves the degradation and replacement of immature ECM such as EDA fibronectin and collagen type III with collagen I, the organization of collagen I fibers into bundles, and the apoptosis of a variety of cell types at the wound site [3, 5, 15, 18, 20, 33]. Together, these changes result in the contraction of the wound and the formation of acellular scar tissue. The remodeling process can continue indefinitely, and scar tissue does not achieve the strength of intact uninjured skin [3, 15].

## Developmental signaling pathways in mammalian wound healing

### Wnt and β-catenin signaling

Wnts are secreted glycoproteins that are important in many fundamental cellular processes during development and in the maintenance of homeostasis in the adult (reviewed in references [34–37]). Wnt ligands signal through the canonical or noncanonical Wnt signaling pathways, depending on the context. The canonical Wnt signaling pathway is mediated through β-catenin, while the noncanonical Wnt signaling pathways, such as the planar-cell-polarity and the Wnt-calcium pathway, are transduced by alternate effectors independently of β-catenin [35, 38]. Here we focus on the canonical Wnt signaling pathway and its key mediator, β-catenin (Fig. 1b; Table 2).

**Table 2:** Comparative summary of the participation of developmental signaling pathways in mammalian skin development and repair

Signaling pathway	Skin compartment^a^	Skin development	Skin repair
Wnt/β-catenin	Dermis	Development of the dermis [41, 43, 44]	Reconstitution of the dermis: fibroblast numbers, cellularity; fibroblast behavior; matrix production [53, 55, 56, 58, 60, 62]
	Epidermis and associated structures	Development and morphogenesis of hair follicles [24, 26, 46–48]	Regeneration of hair follicles in large wounds [7]
TGF-β1	Dermis	Role to be deciphered; expressed in developing dermis [217, 218]	Reconstitution of the dermis; fibroblast proliferation and behavior; matrix production; myofibroblast formation; wound contraction [87–89, 91, 92, 100–102, 107]
	Epidermis and associated structures	No significant role in hair follicle development [84]	Inhibitory role in re-epithelialization [92, 102, 106]
Notch	Dermis	Role to be deciphered	May be involved in dermal reconstitution: macrophage behavior [130]; angiogenesis [129–131]; effect on matrix [130]
	Epidermis and associated structures	Epidermal differentiation [26, 123–125]	Role to be deciphered
Sonic hedgehog	Dermis	Role to be deciphered; in non-mammals (chick embryo), it does not induce differentiation of dermatome (precursor of dermis) [219]	May be involved in dermal reconstitution: effects on matrix, vascularity, and cellularity [140, 141]
	Epidermis and associated structures	Development and morphogenesis of hair follicles [46, 132, 134, 135, 138]	Present in regenerated hair follicles [7]; not expressed by wound keratinocytes [142]

^a^“Epidermis and associated structures” includes the hair follicle and its associated structures (such as the dermal papilla).

β-Catenin is a transcriptional coactivator that associates with other transcription cofactors, such as the T cell factors/lymphoid enhancer factors, to modulate gene expression in a cell type-specific manner (reviewed in reference [36]). In the absence of Wnt signaling, β-catenin is targeted for proteasomal degradation via N-terminal serine and threonine phosphorylation by a cytoplasmic destruction complex that includes the factors casein kinase I, axin, adenomatous polyposis coli protein, and glycogen synthase kinase (GSK) 3β [34–37]. GSK3β is a crucial signaling hub that lies downstream of Wnt, growth factor, and integrin signaling pathways, among others [39], and its activity, and thus that of β-catenin, can also be regulated by these various pathways. Canonical Wnt signaling is initiated by Wnt binding to membrane Frizzled and low-density lipoprotein receptor related protein 5/6 (LRP 5/6), which stimulates the downstream signaling mediator, Dishevelled [34–37]. Dishevelled in turn stabilizes β-catenin by inhibiting the activity of its destruction complex [35], leading to increased cytoplasmic and nuclear levels of β-catenin. In addition to its role as a signaling molecule, β-catenin functions as a structural protein and forms a component of the adherens junctions that mediate cell–cell contacts [34, 40]. It can be freed from this physical association by phosphorylation at specific tyrosine residues [40].

Wnts are important mediators of skin development, participating in various processes from development of the dermis to the formation of skin appendages, such as hair (reviewed in reference [41]). Dermis at different sites in the body, such as the dorsal and ventral dermis, has different origins, and dermal development precedes that of the skin appendages [41, 42]. Canonical Wnt/β-catenin signaling is required for the specification of both murine dorsal [43] and ventral [44] dermis. In murine ventral dermis formation, β-catenin also plays a role in the survival of early ventral dermal progenitor cells [44]. Later in embryonic development, epithelial–mesenchymal interactions lead to the formation of the hair follicle from the epidermal placode, the precursor of the hair follicle [24, 41, 45]. Wnt/β-catenin is essential for hair follicle development during embryogenesis [24, 26, 46–48]. In addition, it is important for differentiation of hair follicles postnatally [24, 26, 46, 49, 50].

Analogous to its function in skin development, Wnt and/or β-catenin signaling plays an important role in various aspects of cutaneous wound repair. As discussed below, it is involved in the construction of epithelial structures and in the reconstitution of the dermal compartment, where β-catenin is a prominent regulator of fibroblast behavior.

Cutaneous wounds express various Wnts during the early phases of healing, with transcripts from Wnts 1, 3, 4, 5a, and 10b present in murine whole cutaneous wounds up to 7 days after wounding [51]. In the epithelium, Wnt10b protein can be detected in migrating epithelial cells up to 3 days after wounding, while WNT 4, 5a, and 10b localize to hair follicles [51]. Wnt 4 is expressed in the dermis, although the time-course of its expression has been variously detected in different studies up to 30 h after wounding [52] and up to 7 days after wounding [51].

Wnt signaling is crucial to the regeneration of hair follicles following injury—a process which has been documented only in large healing wounds [7]. The concept that wound healing recapitulates embryonic development is illustrated by the interesting finding that the source of new follicles is not cells within the hair follicle stem cell niche [7]. This suggests that injury can reprogram or endow other epidermal cells with stem cell or embryonic properties [7].

The role of Wnt/β-catenin signaling in wound re-epithelialization is beginning to be unraveled. Primary keratinocytes harvested from mice expressing conditionally ablated or stabilized β-catenin alleles do not display significant differences in migratory capacity in in vitro experiments [53]. An inhibitory effect of β-catenin on re-epithelialization has been suggested by a study that observed enhanced epidermal nuclear accumulation of β-catenin at the edge of chronic ulcers [54], and found that pharmacological stabilization of β-catenin inhibited keratinocyte migration in culture [54]. Though these studies suggest that β-catenin does not promote keratinocyte migration in vitro, further research is needed to elucidate whether β-catenin or Wnt signaling actively influences re-epithelialization of wounds in vivo.

β-Catenin is an important regulator of fibroblast behavior during the proliferative phase of dermal wound repair. β-Catenin protein levels and transcriptional activity are elevated in dermal fibroblasts during the proliferative phase of healing in murine cutaneous wounds and return to baseline during the remodeling phase [55]. Human wounds similarly show increased expression of β-catenin and its target genes, such as fibronectin and MMP7, during the proliferative phase [56]. Increased β-catenin expression during wound repair has also been noted in other species, such as in the horse [57]. The relative level and activity of β-catenin contributes to the dermal wound phenotype, with high β-catenin levels and activity leading to an enlarged, hypercellular dermal compartment, and low levels of β-catenin associated with a smaller and less cellular dermal compartment [53]. This was demonstrated using mouse models in which β-catenin can be conditionally stabilized or ablated [53]. Similar to the hyperproliferative dermal healing response that is observed in conditionally β-catenin stabilized mice [53], mice with a fibroblast-specific conditional deletion of GSK3β show elevated β-catenin levels and a rapid and fibrotic wound healing response, that includes increased dermal collagen deposition, scarring, and myofibroblast formation [58]. The mechanism was found to involve a β-catenin-dependent pathway [58, 59]. It has been recently shown that knockdown of β-catenin in *Pax7*-expressing cells in murine wounds results in a smaller scar size with fewer dermal fibroblast-like cells [60]. In fact, approximately 25 % of dermal fibroblast-like cells in healing wounds in mice are derived from *Pax7*-expressing muscle progenitor cells that exhibit activated β-catenin signaling [60]. While increased β-catenin activity during the proliferative phase is crucial for successful wound repair, prolonged or aberrant β-catenin activity beyond the normal parameters of healing contributes to excessive fibrosis and scar formation. Indeed human hypertrophic scars and keloids exhibit elevated β-catenin levels [56, 61].

Interestingly, while Wnt ligands may participate in stimulating dermal β-catenin during wound repair, Wnt signaling is not crucial for maintaining elevated β-catenin levels during the proliferative phase of cutaneous healing [62]. This has been demonstrated in mice treated with an adenovirus expressing the Wnt signaling inhibitor Dickkopf (DKK1, which binds LRP6/Arrow [63]), which did not show a significant decline in β-catenin protein levels during the proliferative phase of skin wound healing [62], in contrast to the situation in bone repair [64]. This suggests that other factors play a role in regulating β-catenin levels during the proliferative phase of healing. Indeed, β-catenin levels in fibroblasts can be stimulated by growth factors, such as TGF-β1 [53, 65, 66], that are released during the early stages of wound repair. Furthermore, β-catenin activity in dermal fibroblasts is regulated by ECM components, such as fibronectin, which activate β-catenin through a GSK3β-dependent, β1 integrin-mediated pathway [62]. Integrins, which span the cell membrane, are one of the major pathways of communication between the ECM and the cell interior [67, 68]. During the proliferative phase of wound repair, EDA fibronectin-deficient mice display reduced β-catenin activation, fewer fibroblasts, and decreased wound strength compared to wild-type littermates [62]. These characteristics are rescued by genetic activation of β-catenin using an EDA fibronectin-deficient mouse cross that also expresses conditionally stabilized β-catenin, or through pharmacological stabilization of β-catenin using lithium chloride [62]. This suggests that fibronectin regulates fibroblast behavior through a β-catenin mediated mechanism during wound repair [62]. ECM regulation of β-catenin during wound healing has also been suggested by the finding that wounds created by the novel Picsecond IR laser, which causes minimal tissue or ECM damage compared to traditional surgical lasers, show decreased β-catenin and TGF-β pathway activation compared to wounds created using a surgical scalpel [69].

Mesenchymal progenitor cells also contribute to repair in various contexts [70, 71]. As the differentiation state of mesenchymal progenitor cells is tightly regulated by Wnt/β-catenin signaling [70–72], changes in the level of signaling activity may affect the repair process by impacting differentiation of these cells. For instance, in bone repair, altering β-catenin levels before pluripotent mesenchymal progenitors cells have differentiated impairs healing [64]. In contrast, activating β-catenin levels once progenitors have committed to the osteoblast lineage has a positive effect on repair [64]. The impact of Wnt/β-catenin signaling levels on mesenchymal progenitor differentiation is also illustrated in the context of myocardial healing: in an implantation model of myocardial repair, inhibition of Wnt (and bone morphogenetic protein, BMP) signaling using secreted frizzled-related protein-2 prevents differentiation and apoptosis of bone marrow-derived mesenchymal progenitor cells and improves their engraftment [73].

β-Catenin and Wnt signaling are intrinsically involved in the formation of the dermis and of epidermal structures, both during wound repair and during skin development. It will be interesting to elucidate whether non-Wnt activators of β-catenin, such as ECM proteins and growth factors, modulate β-catenin during skin development as they do during healing. In turn, the role of Wnt/β-catenin signaling in terms of epidermal–mesenchymal interactions during wound repair must be further explored. These reciprocal interactions play a significant role during embryonic skin development [41, 45], but have not yet been as thoroughly investigated during wound healing. A recent study demonstrated that adult dermis can be reprogrammed to regain the characteristics of neonatal dermis in response to epidermal stabilization of β-catenin [74], raising the issue of whether similar or reciprocal interactions could take place during wound repair. In that study, the remodeled dermis was characterized by a downregulation of ECM genes, expression of immature collagens, and increased proliferation of fibroblasts, and these responses were found to originate from fibroblasts in the vicinity of the sebaceous gland [74]. Another example of epidermal–mesenchymal interactions in skin is illustrated by the finding that epidermal hair follicle stem cells secrete the Wnt/β-catenin target, Nephronectin, to form a specialized attachment site for smooth muscle precursors of the arrector pili muscle [75]. Thus, investigating whether Wnt and/or β-catenin participate in epithelial–mesenchymal interactions during wound repair is an important direction for future research.

### TGF-β pathway

Numerous growth factors and cytokines participate in wound repair. These include the TGF-βs, PDGFs, epidermal growth factors (EGFs), fibroblast growth factors (FGFs), vascular endothelial growth factors (VEGFs), and various proinflammatory cytokines, such as the interleukins (reviewed in references [76, 77]). These factors are released by various cell types as well as from sites of sequestration in the disrupted ECM [77]. In this review, we focus on TGF-βs, which are one of the major cytokine mediators of cutaneous wound healing.

TGF-βs comprise the TGF-β1, TGF-β2 and TGF-β3 isoforms, and are part of the TGF-β superfamily that also includes BMPs, among others [10, 78]. During wound healing, TGF-βs are secreted by various cell types, including macrophages and fibroblasts, and they are also released from storage sites in the disrupted ECM [77, 79]. TGF-βs are secreted as inactive precursors bound to the latency-associated and latent TGF-β binding proteins [10, 79]. They are subsequently activated by the action of proteases or through conformational changes induced by integrins in response to cell traction forces [10, 79]. TGF-βs exert cell-specific effects by binding to serine/threonine kinase TGF-β receptor I and TGF-β-receptor II heterodimers, leading to the activation (phosphorylation) of Smads 2 and 3, their association with the common mediator Smad 4, and the subsequent translocation of this complex to the nucleus where they may associate with other transcription cofactors to modulate gene expression [10, 11, 78–80]. Smad 7 is an antagonist to Smad 2/3-mediated signaling [10, 11, 78–80]. Mechanisms of TGF-β signaling that do not involve Smads also exist, including mitogen-activated protein kinase, Akt, extracellular signal-regulated kinase, TGF-β-associated kinase I, and small GTPases, among others [81–83].

Several members of the TGF-β superfamily, including TGF-βs and BMPs, are involved in the development of skin and/or skin appendages, such as hair follicles [78]. Studies involving mice deficient in different TGF-β isoforms have shown that TGF-β2 is required for murine hair follicle development, while TGF-β1 and TGF-β3 do not contribute significantly to this process [84]. In turn, addition of exogenous TGF-β1 to embryonic skin explants has an inhibitory effect on hair follicle development [84]. Using a keratinocyte-specific Smad7-overexpressing mouse model, it was found that Smad-7 can interact with β-catenin to influence both embryonic and adult hair follicle morphogenesis by promoting its degradation via recruitment of the E3 ligase, Smurf 2 [85]. However, the relevance of this mechanism to hair follicle formation under normal physiological conditions must be further explored.

The TGF-β pathway is recognized for its ability to alter the pace of healing. TGF-β1 is characterized as a fibrosis- and scar-promoting factor, while TGF-β3 has been associated with antiscarring actions [6, 86]. For instance, Puolakkainen et al. [87] found that treatment of wounds in aged rats with topical TGF-β1 improved several aspects of dermal healing, including ECM deposition and fibroblast influx and proliferation. TGF-β1 application also improved angiogenesis, inflammatory cell infiltration, and epithelial closure of the wounds [87]. Indeed, TGF-β1 signaling plays a role in many fibrotic effects associated with wound repair, including proliferation of fibroblasts [87, 88], synthesis of ECM components such as collagen I [89] and fibronectin [89, 90] by fibroblasts, transition of fibroblasts to a myofibroblast wound phenotype [91], and contraction of the wound [92]. Some of the fibrotic effects of TGF-β in wound fibroblasts may be mediated through the matricellular protein, connective tissue growth factor (CTGF or CCN2) [93–97]. CCN2 is upregulated in response to TGF-β signaling in fibroblasts during fibrotic conditions such as wound repair, and it also cooperates with TGF-β to mediate some of its effects, such as regulation of collagen expression [93, 94, 97–99].

In vivo animal models, particularly genetically modified mice, have contributed much of our understanding of the fibrotic mechanisms of the TGF-β pathway during wound repair. Mouse models deficient in TGF-β pathway signaling components tend to exhibit defects in dermal healing. For example, the dermis of *Smad 3* knockout mice displays fewer fibroblasts, reduced ECM deposition, and a smaller wound area than control mice [100]. Similarly, wounds in *Tgfβ1* ^−/−^ *Scid* ^−/−^ mice heal with a thin and disorganized dermis, and exhibit delays in all phases of wound healing [101]. Both studies also showed a deficiency in inflammatory cells in the healing wounds [100, 101]. More recent studies have utilized fibroblast-specific deletions of TGF-β receptor II (TGF-βRII) to examine its effect on dermal healing [92, 102]. These studies showed that ablation of fibroblast TGF-βRII causes defects in the dermal ECM, such as a reduction in granulation tissue formation, increased scar size [102], and diminished collagen deposition [92]. Fibroblasts cultured from TGF-βRII knockout mice also display cytoskeletal abnormalities, and reduced contractile abilities [92]. Thus, various mouse models clearly demonstrate that TGF-β signaling is crucial in mediating the fibrotic mechanisms needed to restore the dermis during adult cutaneous wound repair.

While the profibrotic effects of TGF-β1 suggest that it may promote re-epithelialization by stimulating matrix formation for keratinocyte migration, TGF-β1 also has an inhibitory effect on keratinocyte proliferation [103], suggesting that it may impair this aspect of re-epithelialization. In keeping with these different effects of TGF-β1 on keratinocytes, contrasting roles for TGF-β pathway involvement in re-epithelialization have been described in the literature. For example, topical TGF-β1 administration promotes epithelial wound closure in rats [87], and this is complimentary to the delayed re-epithelialization observed in double mutant *Tgfβ1* ^−/−^ *Scid* ^−/−^ mice [101]. Similarly, the wounds of α3β1 integrin-deficient mice display a lag in re-epithelialization which is caused by a suppression of the response to TGF-β1 signaling in keratinocytes via the upregulation of inhibitory Smad 7 [104]. In contrast to studies which have shown a positive role for the TGF-β1 pathway in epidermal closure, other research has demonstrated a negative influence of TGF-β1 signaling on re-epithelialization. For example, deletion of Smad3 in genetically engineered mice results in accelerated epidermal closure and proliferation [100], and TGF-β antagonist treatment of porcine burn wounds increased the number of wounds that healed with complete re-epithelialization [105]. Transgenic mice that overexpress the inhibitory Smad, Smad-7, in the epidermis also exhibited more rapid wound closure, increased keratinocyte proliferation, and in vitro keratinocyte migration than controls [106].

Because TGF-β1 stimulates wound contraction [92, 107], it is possible that some of the seemingly contrasting roles for the TGF-β pathway in re-epithelialization could be caused, in part, by effects on wound contraction being interpreted as epithelialization. Furthermore, different TGF-β ligands and/or pathway components were manipulated in various studies, which may in turn result in different downstream effects. For instance, manipulation of a signaling mediator, such as Smad, affects the signaling of various upstream members of the TGF-β superfamily, and thus influences the function of numerous pathways simultaneously. Crosstalk between different cell compartments, or the relative balance of other signaling mediators, may also be affected by different experimental conditions across studies. In fact, the ultimate effect of TGF-β on re-epithelialization may be determined by the balance of a complex array of factors and conditions at the wound site.

TGF-β signaling during wound healing involves crosstalk between the dermis and epidermis to influence the healing outcome [108]. For example, while fibroblasts release growth factors such as TGF-β that affect keratinocyte behavior [103], keratinocytes can in turn downregulate TGF-β expression by fibroblasts [109]. An influence of epidermal TGF-β signaling on dermal healing is also suggested by a recent study in transgenic mice that over-express Smad7 in keratinocytes [106]. Cutaneous wounds from these mice exhibit altered temporal expression of dermal collagen compared to control mice, and effects on angiogenesis and inflammatory cells were also observed [106]. In turn, mouse models with a fibroblast-specific deletion of TGF-βRII show changes in re-epithelialization, suggesting that dermal TGF-β signaling influences epidermal behavior during wound repair [92, 102]. For example, Denton et al. [102] and Martinez-Ferrer et al. [92] both observed increased keratinocyte proliferation in fibroblast-specific TGF-βRII knockout wounds, and Martinez-Ferrer et al. [92] also reported a hypertrophic epidermis with increased keratin 5 expression. While one study showed a faster rate of re-epithelialization in fibroblast-specific TGF-RII knockout wounds during the early phase of healing [92], another found that the two groups of mice exhibited similar epithelial closure by the proliferative phase of healing [102]. These data support the concept that there is crosstalk between the two skin compartments during wound repair, and suggests that TGF-β signaling in fibroblasts may normally exert some inhibitory effects on keratinocyte behavior during healing.

While TGF-β1 stimulates the fibrotic effects necessary for dermal healing, excessive TGF-β1 activity can lead to the formation of hypertrophic scars, in part through mechanisms mediated by β-catenin [53, 65] or CCN2 [110]. Indeed, TGF-β1 signaling has long been noted for its association with inflammation and fibrosis following injury. One of the differences between adult and scarless fetal wound healing is the near absence of TGF-β1 in the early fetal repair process [4, 111]. The scar-promoting effects of TGF-β1 are exemplified by the scarring reminiscent of adult wound repair caused by the application of TGF-β1 to embryonic skin wounds [111]. In fact, scar-free embryonic wounds in rats have lower TGF-β1 and higher TGF-β3 levels than the scar-forming adult-like wounds that occur in late gestation [6]. The effects of hypoxia on TGF-β3 [112, 113] may account for the increased TGF-β3 levels in fetal versus adult healing. The application of TGF-β3 has been shown to reduce scarring in a rodent wound model [86], although some reports dispute the antiscarring abilities of this molecule [114]. TGF-β3 has been reported to reduce scar formation through effects on matrix production, cell movement and inflammatory mediators [115]. However, the precise molecular mechanisms involved are not clear. One possibility is raised by the finding that human serum (which contains TGF-β3) promotes epithelial migration, while plasma (which does not contain detectable TGF-β3) encourages dermal fibroblast migration [116]. It was suggested that the transition between these media (and thus exposure to soluble TGF-β3) during wound healing influences the motility of different cell types [116]. It is conceivable that a related mechanism might operate during fetal wound healing to differentially influence the behavior of cells in different skin compartments exposed to TGF-β3. However, research also suggests that the ability of fetal wounds to heal without scarring is an inherent property that does not depend on the fluid environment of the embryo [9, 117]. The detailed mechanisms whereby TGF-β3 may exert antiscarring actions await further investigation. Furthermore, as noted above, whether TGF-β3 can act as a potential antiscarring agent is controversial. While a pharmaceutical formulation of TGF-β3 has shown promise in improving the appearance of scars in phase II clinical trials [118], the drug did not meet some of the expected endpoints in phase III clinical trials [119].

Different components of the TGF-β signaling pathway seem to be important in skin development and in postnatal wound repair. For example, while TGF-β1 is a crucial component of the fibrogenic aspect of postnatal healing, it does not appear to play a significant role during embryonic skin morphogenesis. In contrast, other TGF-β signaling molecules or mediators, such as TGF-β2, participate in mediating certain aspects of skin development, such as hair follicle formation [78, 84].

### Notch pathway

The Notch signaling pathway is a regulator of epidermal differentiation, both in the maintenance of skin homeostasis in the adult, and in embryonic development of the epidermis [26, 120, 121]. It also plays a major role in other developmental and physiological processes, including angiogenesis and vascular maintenance [122]. Notch is known to engage in crosstalk with the Wnt/β-catenin and Hedgehog pathways [120], two other signaling pathways that participate in skin development and homeostasis. Notch signaling requires an interaction between a transmembrane ligand and the transmembrane Notch receptor on two adjacent cells [120–122]. In mammals there are four Notch receptors, and their ligands are members of the Delta-like and Jagged families [120–122]. Ligand binding causes sequential proteolytic cleavage of the Notch receptor, resulting in the liberation of the Notch intracellular domain (NCID) from the cell membrane and its translocation to the nucleus where it associates with RBP-J and other cofactors to modulate the transcription of target genes such as the Hes/Hey transcriptional repressors [120–122].

During skin development, Notch signaling regulates epidermal differentiation to ensure correct stratification of the epidermis [26, 123, 124]. Interestingly, cilia are involved in stimulating the Notch pathway during epidermal development, as demonstrated by the finding that mice lacking epidermal cilia show attenuated Notch signaling and impaired epidermal differentiation [125]. Some components and/or modulators of Notch signaling are expressed during development of dermal portions of the skin: Delta1 [126, 127] and Lunatic Fringe [127] are expressed in dermal condensates [126, 127], while Notch2 has been identified in the adjacent mesenchyme [127].

Research has suggested that the Notch pathway participates in wound repair, and that it is required for proper healing. Thelu et al. [128] described expression of Jagged1 and Notch1, as well as the signaling modulator, Lunatic Fringe, in healing epidermis of wounded human skin grafted onto mice [128]. Later studies examined the role of the Notch pathway during wound repair. Transgenic mice expressing a Notch antisense sequence that causes a 50 % reduction in Notch protein, exhibited delayed healing as assessed by surface wound size [129]. Mice treated with the Notch ligand, Jagged, show accelerated wound closure (as assessed by surface wound size) suggesting that these effects are mediated by the Notch pathway [129]. In vitro scratch wound assays with cultured cells, using a Notch inhibitor or activator, suggest that part of the mechanism may involve promigratory effects of Notch on fibroblast and vascular endothelial cells [129]. In turn, Outtz et al. [130] found that Notch1 hemizygous (heterozygous) mice exhibit increased collagen deposition and vascularity in healing wounds. The participation of Notch signaling in angiogenesis during wound repair was also suggested by Caiado et al. [131] following in vitro studies where the Notch pathway regulated the matrix-adhering and angiogenic properties of bone marrow-derived vascular precursor cells. Injection of such cells into mice caused a reduction in the size of healing wounds and also increased the density of wound microvessels, whereas mice that were injected with cells treated with a Notch inhibitor did not exhibit these effects [131].

Interestingly, a potential role for Notch signaling in the wound inflammatory response, a process that does not exist during development, has recently been elucidated. Outtz et al. [130] utilized Notch1 hemizygous (heterozygous) mice, as well as mice with a myeloid-specific deletion of Notch1, to study the genetic effects of Notch1 deficiency on macrophage function during wound repair. Both mouse models exhibited reductions in macrophage recruitment and in the expression of various inflammatory cytokines and growth factors commonly secreted by macrophages [130]. The data suggest a role for Notch in regulating macrophage behavior during the inflammatory phase of healing [130]. In particular, in vitro and in vivo studies showed that Notch1 can modulate VEGF1 expression by macrophages [130]. Interestingly, wounds from mice with a myeloid-specific deletion of Notch1 did not show changes in the wound phenotype in terms of collagen deposition or the number of blood vessels, in contrast to mice with a general Notch1+/− deletion, where these parameters were increased [130]. This suggested that cells other than macrophages are involved in mediating some of the effects of Notch1 on the wound phenotype [130].

There is evidence that the Notch pathway participates in wound repair, and that it may be involved in several aspects of healing, such as angiogenesis, matrix production, and inflammation. However, the role of Notch signaling in wound healing has not been as extensively studied as have some other pathways, and there are still many questions to explore. Some of the suggested roles for Notch signaling in healing are quite different from its function in skin development. For example, Notch’s involvement in macrophage behavior during the inflammatory phase of wound repair is unique in the sense that this function would not be utilized during embryonic development. In addition, Notch is well known as a regulator of epidermal differentiation of skin [26, 120, 121], but its role in this process during the re-epithelialization of healing wounds is relatively unexplored. In keeping with this, any influence of Notch on the behavior of various cell types, such as keratinocytes and fibroblasts, during healing must also be investigated. Thus, there are many interesting avenues of potential future research with regard to the involvement of Notch signaling in cutaneous wound repair.

### Hedgehog pathway

The Hedgehog signaling pathway is involved in many aspects of embryonic development, including skin morphogenesis and angiogenesis [132, 133]. In skin development, it plays a significant role in the development of hair follicles and associated structures such sebaceous glands and the dermal papilla, as well as in the regulation of hair follicle and epidermal stem cells in the adult [46, 132, 134–138]. Hedgehog, Wnt/β-catenin, and Notch signaling interact in many of these processes. Three Hedgehog proteins exist in mammals, termed Sonic Hedgehog (Shh), Desert Hedgehog (Dhh), and Indian Hedgehog (Ihh) [132, 139]. Here we focus on Shh signaling. Shh binds to the transmembrane protein and tumor suppressor, PTCH, to de-repress the inhibition of Smoothened, resulting in activation of the Gli transcription factors and the regulation of downstream target genes [132, 139].

While the Shh pathway’s involvement in skin development has been extensively studied, much less is known about its role in wound repair. Nevertheless, several recent studies have begun to address this issue and suggest that the Shh pathway may have the ability to modulate several aspects of wound healing, such as dermal repair, and wound vascularization [140, 141]. Shh is present in regenerated hair follicles following wound healing [7], but it is not expressed at detectable levels in wound epidermis or in the keratinocytes that contribute to re-epithelialization [142]. A study that used a reporter mouse expressing LacZ upstream of *Ptc1* to examine Shh pathway activation in murine wounds, found Patch1 expression in hair follicles close to the wound site and in the dermis adjacent to the hair follicles [140]. The authors then tested the effects of Shh on wound repair in diabetic mice using methylcellulose pellets that contained a human Shh-expressing plasmid. Topical application of the plasmid to mouse wounds stimulated Shh expression in keratinocyte-like and fibroblast-like cells [140]. Interestingly, diabetic mice treated with Shh exhibited improvements in re-epithelialization and dermal healing, including a larger and more collagen-rich dermal compartment, compared to control mice [140]. The wounds of Shh-treated mice also exhibited increased cellularity and vascularity [140]. The ‘fibrotic’ effects observed in Shh-treated wounds may be caused by enhanced fibroblast activity, as Shh stimulated proliferation of primary dermal fibroblasts in vitro [140]. Further, it was found that Shh treatment increases both VEGF expression and the recruitment of bone marrow-derived endothelial progenitor cells to wound vasculature, suggesting a possible mechanism for the increased vascularity in Shh-treated wounds [140]. Complimentary to these results, in a more recent study, mice treated with the Shh inhibitor cyclopamine for 30 days after wounding showed delayed wound closure and reduced dermal granulation tissue formation [141]. In addition, wound vascularity and cell proliferation were adversely affected in response to Shh inhibition [141]. Another study also found activation of the Shh pathway in murine cutaneous wounds, and treatment of diabetic mice with topical Shh improved healing by modulating the nitric oxide pathway [143].

Thus Shh has the potential to influence several aspects of wound healing, including dermal tissue repair and vascularization. Additional studies are required to elucidate the mechanisms behind some of these effects and to define the physiological role of Shh in wound repair more precisely, including its function(s) in different cell types. One area of interest would be to determine whether Shh signaling is a participant in epithelial–mesenchymal interactions during wound repair. Importantly, future research on Shh in wound repair should be undertaken using appropriate genetically modified mouse models to compliment the largely pharmacological studies in this area to date. Thus, while the role of Shh in wound repair is a relatively unexplored topic compared to its role in skin development, the evidence suggests that this is a promising area of future research.

## Wound healing across the taxa

All organisms are exposed to environmental cues around them. For a long life, multicellular organisms must maintain their tissue morphology and its function. The environment is riddled with potential sources of harm—from sharp surfaces to infectious organisms searching for routes to penetrate an organism’s body armor. To deal with these threats, multicellular organisms are equipped with a strong waterproof barrier that protects them from physical harm. All multicellular organisms are able to respond to injury and repair their tissue to some extent. For example, upon epidermal injury, a conserved innate immune system functions in both vertebrates and invertebrates [144] to combat infectious microbes. In order to preserve tissue integrity, it is essential that organisms detect the loss of tissue mass, activate the de novo production of cells, and organize those cells into functional tissues. However, there is huge diversity in how the process of healing occurs across the taxa. Some multicellular organisms can regenerate the missing part of the body while others cannot. In spite of this diversity, there are basic principles of healing shared by many multicellular organisms: injury detection, gap closure, cell division activation to supply new cells, cell differentiation, and remodeling of newly formed tissue. Even the earliest branching animals (e.g. the sponge *Oscarella carmela*) express core components of the Wnt, TGF-β, Notch and Hedgehog signaling pathways [145]. In mammals, despite considerable ability for tissue regeneration, large wounds result in the formation of scar tissue instead of a complete restoration of tissue morphology and function [5]. This limited regenerative capacity is partly due to rapid interposition of fibrotic tissue, something that prevents subsequent tissue regeneration, but might be a defensive advantage in preventing harmful microbes [5]. Tissue regeneration in humans, however, is very limited. If injured, only bone, liver and infant finger tips can regenerate [70, 146, 147]. Aging is another determinant for tissue restoration, as animals gradually lose their regenerative capacity as they get older. The diversity in tissue restoration is due to the species, the type of injured tissue, and the age of the animal. It seems that during vertebrate evolution, some of the regenerative ability of lower multicellular organisms was inactivated.

As noted, core components of the Wnt, TGF-β, Notch and Hedgehog signaling pathways are expressed in sponges and higher organisms across the taxa [145]. Preservation of signaling pathways across taxa requires a universal way by which multicellular organisms respond to injury and heal their bodies. If we are able to unveil the mechanism of tissue preservation across the taxa, we may open new doors for tissue/organ regeneration in humans, preferably by enhancing our endogenous ability which was inactivated during evolution. A clue may come from the following nonmammalian species that have an impressive ability to regenerate (Table 3).

**Table 3:** Contribution of developmental signaling pathways to tissue healing (regeneration) in selected nonmammalian species

Animal group	Wnt/β-catenin	TGF-β	Notch	Hedgehog
Planarians	Tail regeneration			Tail regeneration
Fly	Dorsal closure	Dorsal closure		
Fish	Blastema formation			Fin regeneration
Amphibians	Tail regeneration, limb regeneration	Tail regeneration	Tail regeneration	Tail regeneration

Empty cells indicate that there are no conclusive studies that support involvement of the indicated signaling pathway

### Planarians

Planarians are a group of nonparasitic flatworms. They lack complex organs but can fully regenerate [148]. A planarian split lengthwise or crosswise will regenerate into two separate individuals. Planarian regeneration involves changes in preexisting tissues and the formation of an outgrowth. It starts with the formation of a mass of proliferating cells (blastema) at the site of injury. Blastema cells become organized into tissue and regenerate organ systems [148].

Small fragments of tissue (as little as 1 part in 289) [5] can regenerate into entire new animals, including all components of the body. When a planarian is cut transversely, the caudal fragment will regenerate a head and the anterior piece will regenerate a tail. It is has been shown that after injury, Wnt signaling promotes tail regeneration. In the head-versus-tail regeneration experiment, Wnt inhibitor *notum* preferentially expresses at anterior-facing wounds [149], highlighting the essential role of Wnt signaling during planarian regeneration. Planarians depleted of WntP-1 regenerate a head in place of a tail [150]. On the other hand, Hedgehog signaling induces regeneration of a tail instead of a head through the activation of Wnt transcription [151, 152]. This suggests the importance of Wnt/β-catenin and Hedgehog pathways for anteroposterior axis specification during regeneration.

### Fly

Mammalian skin and insect cuticle form a protective barrier that helps prevent dehydration and protect against injury. In the fruit fly *Drosophila*, this barrier has a single-cell layer of epidermal cells that secrete cuticle. This cuticle is equivalent to the stratum corneum in mammalian skin, which consists of dead squamous epithelial cells.

Developments in genetic manipulation have allowed the study of wound healing signaling pathways in fruit flies which may provide new insights into biological processes, including wound healing, in humans. Despite differences in the molecular composition of insect cuticle and mammalian skin, it is noteworthy that some regulatory mechanisms for the development and healing of protective barriers in insects and mammals have been conserved. For instance, a master gene called *grainy head* (g*rh*) activates wound repair genes in the cells surrounding an injury in the cuticle of fly embryos [153]. These wound repair genes then regenerate the injured patch of cuticle. Wounds in mutant flies that lack the g*rh* gene fail to heal. Interestingly, the g*rh* gene is also essential for normal skin development and wound repair in mice. Like their fruit fly counterparts, mice lacking g*rh* have a much more permeable skin than normal mice, and also have deficient wound repair [154]. These two studies signify an imperative concept in wound healing: although the structure of the surface barrier is considerably different across taxa, the signaling pathways that coordinate healing of the barrier are evolutionarily conserved.

Morphological events such as dorsal closure [155] and tracheal fusion [156] during *Drosophila melanogaster* development, have notable similarity to wound repair in humans. Dorsal closure of the *Drosophila* embryo is the best-characterized example of epithelial sheet movement leading to epithelial fusion. It involves migration of the lateral epidermal flanks to close a hole in the dorsal epidermis occupied by an epithelium [157]. Morphogenetic events involving tissue migration and epithelial fusion have been used as models to examine the involvement of developmental signaling pathways in wound repair. Wnt/Wingless (Wg) signaling has been shown to play an essential role during dorsal closure [158]. Moreover, the TGF-β signaling pathway is required for dorsal closure, and acts downstream of the JNK cascade [159, 160]. These signaling pathways regulate cytoskeletal reorganization and cell shape change to orchestrate cell migration for dorsal closure.

### Fish

In some of the fish species, when a major part of the fin, or even the entire fin, is removed, it is regenerated with recovery of its shape and function [161]. Fish fins have been used for analyzing the regeneration process for more than two centuries [162]. Caudal fin regeneration is dependent on the presence of musculature and endoskeleton at the site of amputation [161]. A layer of epidermal cells covers the fin rays and the mesenchymal tissue around them. Immediately after injury, an F-actin purse string is formed in the epithelial cells surrounding the wound gap and quickly contracts to close the epithelial opening [14]. Later, the epithelial cells migrate to make a tight epithelial sealing on the wound gap. Wound closure is complete by this stage. The process of cell proliferation starts and forms a new epidermis (called wound epidermis) to cover the wounded area. As in their mammal counterparts, this epidermis layer is distinguishable from the surrounding epidermis by its thick morphology [163]. Following the formation of the wound epidermis, a mass of proliferating mesenchymal cells forms (again called blastema). The blastema (which is in close contact with the wound epidermis) continues to proliferate until an adequate cell supply is available to replace the lost part. In order to study the developmental signaling pathways involved in healing, different genetic methods or specific biochemical assays have been used. Here, we discuss the role of some of the above-mentioned signaling pathways during the fish healing process.

Amputating the caudal fin in zebrafish stimulates regeneration of the dermal skeleton and re-expression of Shh signaling pathway genes. The essential role of Hedgehog signaling in bone differentiation in the fin-ray has been demonstrated [164] using cyclopamine (an inhibitor of Hedgehog signaling). Exposure to cyclopamine alters bone patterning. FGF receptor 1 (FGFR1) is expressed in mesenchymal cells underlying the wound epidermis during blastema formation and in distal blastemal tissue during regenerative outgrowth. Using SU5402 (an inhibitor of FGF signaling), blastemal cell proliferation is blocked, leading to the prevention of outgrowth during ongoing fin regeneration [165]. This shows the necessity of FGF signaling for zebrafish fin blastema formation and regenerative outgrowth. It is interesting to note that the Wnt/β-catenin signaling acts upstream of FGF signaling [166]. The importance of Wnt/β-catenin signaling has been shown using transgenic fish lines. During zebrafish tail fin regeneration, Wnt/β-catenin signaling is activated and is required for the formation, and subsequent proliferation, of the blastema progenitor cells [166]. Using a dominant-negative form of T cell factor (to transcriptionally block Wnt/β-catenin signaling) or DKK1 (to block Wnt/β-catenin signaling at the ligand level), this essential role of the Wnt signaling pathway was highlighted [166]. Furthermore, MMP inhibitors also inhibit the regeneration process and result in reduced cell proliferation in the blastema [167]. Studies over the last few decades have shown the critical role of some of these developmental signaling pathways during fish regeneration and have advanced our understanding of how activation of these pathways in a timely manner leads to tail fin regeneration.

### Amphibians

Some species of amphibians, a class of vertebrates including frogs and salamanders, have the ability to regenerate amputated appendages through the formation of a blastema. While the origin of blastema cells is unclear, in urodeles, it has been suggested that the blastema is formed by the de-differentiation of nearby cells [168, 169]. This highlights the existence of plastic residual differentiated cells rather than the existence of so-called “reserve stem cells” [170]. However, using a lineage-tracing approach in the salamander, each tissue produces progenitor cells with restricted potential [171]. While this challenges the existence of a unique cell with multipotential regenerative capacity, it nonetheless reveals the fabulous endogenous regenerative machinery that is partly inactivated in humans. Adult urodele amphibians can restore limbs, tails, the lens and retina of the eyes, and heart tissue [172]. This remarkable ability, together with the close relationship between amphibians and mammals, makes amphibians a very attractive model for the study of regeneration.

 The importance of several developmental signaling pathways has been shown in recent studies on *Xenopus*. Wnt signaling inhibition using DKK1 at the beginning of regeneration is sufficient to efficiently inhibit both tail and limb regeneration in *Xenopus* [173]. Likewise, using BIO (a cell-permeable compound that acts as a highly potent ATP-competitive inhibitor of GSK-3α/β [174]) as an enhancer for the Wnt/β-catenin signaling pathway causes blastema cell proliferation to increase and leads to enhanced outgrowth of the regenerating tail [175]. Notch signaling is also necessary for tail regeneration in *Xenopus*. Blocking Notch signaling with the protease inhibitor MG132 (which inhibits the cleavage of the NCID) blocks tail regeneration [176], suggesting involvement of the Notch signaling pathway in tail regeneration. However, considering the nonselective effect of this protease inhibitor, the specific targeting of the Notch pathway using this compound remains to be defined.

In *Xenopus*, the notochord provides a source of Shh to the cells near the amputation site. Therefore, it is conceivable that the availability of Shh in the stump tissues determines the regenerative potential of each amputated site. In support of this, treatment with cyclopamine (the Shh signaling inhibitor) has been shown to prevent tail regeneration in urodeles [177]. However, unlike tail regeneration, cyclopamine-treated regenerating limbs attain a normal length and contain cartilage [177]. This shows that temporal and spatial expression of morphogens during development may give a clue as to which developmental signaling pathway plays the crucial role for the regeneration of each organ. TGF-β signaling has been shown to play a vital role in wound healing of the tail [178]. Amputated animal tails that are treated immediately and continuously with SB-431542 (a TGF-β-specific inhibitor) fail to form a wound epidermis and tail regeneration is blocked [178].

Unlike most amphibians which undergo metamorphosis (from egg to larva and larva to adult form), in a condition called neoteny, the axolotl continues to show larval features throughout its life, retaining external gills and with undeveloped limbs [179]. These paedomorphic axolotls are fully aquatic, sexually mature and fail to completely metamorphose. Moreover, they are capable of flawlessly repairing full-thickness excisional wounds made on their dorsal flank [179]. A study comparing skin regeneration between paedomorphic and metamorphic axolotls (by induction of metamorphosis in adult axolotls) has shown that aquatic (paedomorphic) axolotls re-epithelialize faster, have fewer leukocytes during early inflammation, have less ECM deposition and a faster regeneration (two times faster for complete skin regeneration) [180]. This highlights the relationship between retention of larval characteristics (i.e. paedomorphic axolotls) with the process of regeneration (faster regeneration) as observed in other vertebrates [181]. However, the fact that both paedomorphic and metamorphic axolotls are capable of scar-free healing (with a delay in metamorphic axolotls) implies that ultimately both paedomorphic and metamorphic axolotls are able to revert to the earlier stage of development to regenerate, suggesting an intrinsic capability in cells regardless of their larval stage. Unraveling this intrinsic capability might be the key for scarless skin healing and complete regeneration in humans.

## Clinical aspects of wound healing in humans: perspective

The human skin forms a large physical barrier between the body and its environment. Skin diseases can have many mental and physical effects on the quality and length of life [182]. Likewise, deficient wound healing may impose huge mental and physical burdens on patients. It has been reported that about 15 % of older adults suffer from chronic wounds [183]. Furthermore, every 30 seconds, a lower limb is lost somewhere in the world as result of wound infection [183]. This not only affects the personal life of the individual, but also has a huge economic effect on society. The burden of treating wounds with deficient healing is growing rapidly due to escalating health-care costs, aging populations, and a sharp increase worldwide in the incidence of diseases such as diabetes. In the US alone, the collective health-care cost of chronic wounds is estimated at $25 billion annually [184]. The annual wound care products market reached $15 billion in 2010 [183]. These factors all impose a great cost on society, and emphasize the necessity for better management of deficient wound healing. One strategy is to develop therapies that manipulate the developmental signaling pathways discussed above in an attempt to recapitulate wound repair observed during fetal healing and the process of regeneration.

While some wound healing complications are due to delayed healing, occurring in patients with diabetes, immunodeficiency, or radiation exposure, some complications are also due to excessive healing, such as hypertrophic and keloid scarring [5]. Such scarring causes morbidity, particularly when the scar is close to a vital organ or if it limits the motion of a joint. Hypertrophic scars mainly occur after major injuries such as burns, leading to poorly structured skin with cosmetic and medical consequences. On the other hand, keloid scars are tough, heaped-up scars that rise quite suddenly above the skin for unknown reasons after a minor injury. They tend to enlarge progressively and occur with a familial tendency [185]. Some keloids arise from a mature scar some years after the initiating events [185]. Several studies have investigated the contribution of molecules in the SMAD-dependent TGF-β signaling pathways at both the mRNA and protein levels in keloids [186–189]. It is intriguing that TGF-β has been shown to upregulate the Wnt/β-catenin pathway in hypertrophic scars and keloids [53, 56, 61]. Moreover, in skin organotypic culture, R-spondin2 (a secreted protein known to be a Wnt/β-catenin signaling agonist [190]) thickens the epidermis, a hyperproliferative phenotype observed in keloids [191]. These studies suggest a role for Wnt/β-catenin signaling in the pathogenesis of keloids. Although no direct involvement of the Hedgehog and Notch signaling pathways in keloid pathogenesis has been suggested, Gli-1 has been shown to be upregulated in keloids [192].

For decades, growth factors were the main focus of research. Several studies have tried to identify the role of growth factors during skin healing, mainly considering wound healing enhancers [193, 194]. This has made growth factors the core of the therapeutic approach for disturbed healing, which has in turn led to several double-blind trials using different growth factors in humans. Some of these trials have shown promising preliminary results, but none of the growth factors has been approved for clinical use [14, 15, 195–197]. A correlation exists between increased amounts of TGF-β and fibroblast hyperproliferation during hypertrophic scarring [66, 198, 199]. On this basis, clinical studies were designed to decrease scar formation using antibodies or molecules directed against TGF-β [200]. For example, early work investigating the application of exogenous TGF-β2 to venous stasis ulcers was promising [201]. Recently, a phase II clinical trial study showed that intradermal TGF-β3 administration following scar revision surgery significantly improved scar appearance compared with a placebo [202]. However as noted, it did not meet the expected endpoints in phase III clinical trials [119]. A TGF-β antagonist increased the speed of re-epithelialization and reduced scar formation and wound contraction in a partial thickness burn experiment in pigs [105]. While the recent trend seems promising, multiple well-designed clinical trials will be indispensable to evaluating the efficiency of TGF-β components for the management of deficient wounds. This is because multiple clinical trials have failed to show the efficiency of TGF-β in the treatment of chronic wounds [76].

To date, only human recombinant PDGF-BB has received FDA approval for the management of limb diabetic ulcers [203], and ulcers treated with PDGF-BB showed only a 15 % greater healing incidence compared to placebo-treated ulcers [203–205]. Moreover, in 2008 the FDA warned of an increased risk of cancer mortality in patients who need extensive treatment with PDGF-BB. There are also some reports on the anti-scarring effects of FGF-2, including in clinical use [206]. Postoperative administration of FGF-2 inhibits hypertrophy and widening of scars without any serious side effects. Indeed, in Japan, FGF-2 is already used clinically. Moreover, there are some reports which highlight the clinical advantage of using EGF during recovery from life-threatening conditions [207].

 In spite of all these advances, these approaches have not led to substantial changes in patient care. It is likely that limited success in single-factor therapies is due to their off-target effects, the difficulty in correctly timing their administration during healing, and the dynamic nature of the wound healing process. In addition, the interactions among growth factors, their receptors, and other ECM components are also critical for the clinical delivery of growth factors. Studies in a diabetic mouse model of chronic wounds have shown that an engineered form of recombinant fibronectin greatly enhances the regenerative effects of administered growth factors [208]. This suggests that combination therapy may tackle some barriers in the management of disturbed wound healing. An alternative approach to wound management would be to use cells that contribute to normal healing. Providing the cells whose contribution in normal wound healing is known together with the correct microenvironmental elements that are important for these cells to thrive, could be a feasible alternative approach in the management of deficient skin healing. Therefore, finding the origin of cells that contribute to normal skin wound healing may open new doors to candidate cell therapy sources, and these cells could potentially be sources of growth factors as well [60].

Approaches that are currently available to optimize wound repair include refinements in surgical technique, nutritional supplementation, and the use of local wound care modalities [209]. Despite these approaches, there has been little progress in the ability to regulate wound size. Surgical advances have helped minimize deficient skin healing following surgery, but scar formation is still just a minimal cost of surgery. Laser technology has evolved over the past few decades and may be useful in the treatment of select scars [210, 211]. However, more research is needed with less bias, better-defined laser settings, better outcome measurement characteristics, and standardized measuring methods to evaluate outcomes in humans. Animal models and in vitro experiments have also shown that lasers can facilitate rapid resolution of cutaneous ulceration, although this has yet to be consistently observed in humans.

The laser was first used as a surgical tool shortly after its invention as an alternative to mechanical surgical tools [212]. As a cutting modality, lasers can perform surgery at the fundamental limit by exploiting the spatial phase coherence of laser radiation to focus sufficient intensity for ablation or cutting at the single cell level. A recent study has shown that, in mouse wound healing, a new laser is able to ablate tissue at the single cell level [69]. Of particular note, the smaller scar size in tissue ablation is accompanied by less Wnt/β-catenin and TGFβ signaling pathway activation [69]. This tackles part of the problem that exists with thermal lasers [213].

Despite all these advances, the ultimate standard for management of deficient skin healing is to prove the relevance of animal wound healing research to humans. Effective therapeutic and preventive strategies depend on our understanding of wound healing, the biology of scar formation, and the cells that contribute to the healing process. This may be achieved by elucidating the molecular pathways whereby adult cutaneous healing differs from embryonic skin development and regeneration. The ultimate goals should be the transformation of fibrotic events into regenerative events in the case of over-scarring, as well as the provision of cells (and cell niche) to regenerate in the case of non-healing. Studying the developmental signaling pathways that are essential for wound healing and manipulating them at the right time during the course of wound healing may be a strategy for tackling some complications. The vast social and economic impact of disturbed wound healing calls for the allocation of more resources to identify biological mechanisms underlying the complexities of wound healing.

## Conclusions and future directions

Here we have discussed the roles of several key developmental signaling pathways (Wnt/β-catenin, TGF-β, Notch, Hedgehog) in mammalian cutaneous wound repair and compared them with their functions in embryonic skin development and regeneration. The Wnt/β-catenin, Notch, Hedgehog and TGF-β families are well characterized as major regulators of developmental processes. The TGF-β pathway in turn is infamous as a mediator of cutaneous healing. Because of the parallels between skin healing and embryonic skin development, a review of the research concerning the roles of these developmental signaling pathways in cutaneous wound repair has the potential to generate new perspectives for these events. Although both skin repair and development are designed to construct skin, they have very different outcomes in mammals. The properties of the scar tissue produced by cutaneous wound repair differ substantially from the skin we are born with. Insights into the relationship between skin repair and development may be harnessed to generate new therapies for treating wound healing disorders. Similarly, understanding the signaling events that regulate healing in species with the ability to completely regenerate their damaged tissue may suggest strategies for improving wound healing outcomes in humans. To achieve these goals, future research should aim to elucidate the mechanisms which differentiate mammalian skin repair from skin development and regeneration.

The inflammatory response constitutes one of the key differences between cutaneous wound repair and embryonic skin development in mammals. While the inflammatory response is crucial to protect the organism from infection and further harm, it is also a major factor in the pathogenesis of scar formation. As discussed above, the actions of inflammatory cells and their cytokines released during wound repair contribute to fibrosis and the formation of scar tissue. The aim of clinical therapies should be to achieve an optimal balance between the protective function of inflammation and the prevention of excessive scar tissue formation. This may be achieved by enhancing our understanding of how and when developmental and other signaling pathways interact during inflammation and in the transition to the proliferative phase of healing. For example, some growth factors and ECM components are present at different concentrations in scar-less fetal repair versus adult healing [4, 6, 9]. Further insight into the mechanisms that regulate the transition in the wound milieu between the fetus and adult may allow manipulation of signaling mediators to mimic the embryonic environment.

In general, the developmental signaling pathways discussed here have been more extensively investigated in the context of developmental biology than in wound repair. However, it is clear that many of these pathways play parallel or similar roles in both processes. As these signaling pathways are involved in complex epithelial–mesenchymal crosstalk during development, it is imperative to further investigate such interactions between different cellular compartments during cutaneous wound healing. Related to this is the concept of crosstalk between cells and their microenvironment or niche [16], a subject of intense interest, particularly in the fields of developmental biology, stem cell, and cancer research. As a recent example from our laboratory shows in terms of the influence of fibronectin on fibroblast behavior [62], these interactions also take place during wound repair. Directions for future research thus include further investigating crosstalk between different cell types (including skin stem cells) and compartments, and between cells and the ECM, during the wound repair process. The wound is a dynamic environment and achieving a complete understanding of the nature of these relationships, which may occasionally be fluid or transient, is certainly a challenging task. However, it is also an important area of investigation if we are to fully understand the mechanisms that regulate skin repair and those that regulate development.

Finally, while we have discussed only a few key developmental signaling pathways in this review, a multitude of other signaling mediators participate in wound repair, development, and regeneration, some of which may not yet have been identified. Insight into the identities of the molecules and pathways that may account for differences between postnatal and fetal healing has been aided by high-throughput approaches, such as expression array analysis [214, 215]. Although gene expression studies are valuable in identifying candidates, they should be combined with proteomics approaches to uncover possible variations in protein synthesis or modification between subjects. The role of microRNAs in regulating wound repair [216] is another emerging topic of exploration that may elucidate some of the regulators in adult and fetal healing. While such mass-throughput approaches certainly represent a valuable means of filtering information and identifying possible targets for further analysis, it is important that the interactions of candidate molecules and signaling pathways be validated in vivo. The complex and dynamic wound environment cannot be completely recapitulated in vitro, but can be most thoroughly examined in its natural in vivo setting where all of the required factors, both known and unknown, are present.

The intricate and dynamic nature of the wound environment suggests that successful therapies for treating wound healing disorders will not rely upon a single all-encompassing agent, but will likely require a multitude of factors administered at different periods during the wound healing process. Identifying the relationships between developmental signaling pathways in adult wound repair and fetal skin development and/or regeneration will certainly propel the research community closer to this goal, and is a fruitful area of future investigation.
